# Enhancement of Morpho‐Physiological, Physiological, and Antioxidant Capacity of Saffron (*Crocus sativus* L.) Under Water Deficit Using ZnO Nanoparticles and Magnesium Sulfate: Implications for Saffron Yield

**DOI:** 10.1002/fsn3.72236

**Published:** 2026-08-11

**Authors:** Laila Turki Fadala, Seham Zaben, Ekhlas Meteab Ahmed Marir, Heidar Meftahizade

**Affiliations:** ^1^ Department of Horticulture and Landscape Engineering, College of Agriculture and Marshes University of Thi‐Qar Nasiriyah Iraq; ^2^ Department of Biology, College of Education for Pure Sciences University of Thi‐Qar Nasiriyah Iraq; ^3^ Department of Horticulture and Landscape, College of Agriculture University of Diyala Diyala Iraq; ^4^ Department of Horticultural Sciences, Faculty of Agriculture & Natural Resources Ardakan University Ardakan Iran

**Keywords:** antioxidant activity, drought stress, nanoparticle, saffron, water deficiency

## Abstract

Water deficit stress is one of the main plant growth and productivity limitation factors. In current study, the *Crocus sativus* plants were exposed to three irrigation regimes (FC 100%, FC 75%, and FC 25%) combined with ZnO‐NPs (0, 25, and 50 mg L^−1^) and MgSO_4_ (0, 1.5, and 3 g L^−1^). Growth, flowering, physiological, and biochemical characteristics were evaluated in a split‐plot experiment based on randomized complete block design (RCBD) with three replicates during 2023–2024. The results demonstrated that severe drought stress markedly reduced leaf dry weight, flower weight, flower number, stigma dry weight, and corm production, while increasing ionic leakage, catalase activity, and proline accumulation. The combined application of ZnO‐NPs and MgSO_4_ improved relative water content, photosynthetic efficiency, antioxidant activity, vegetative growth, and flowering performance under full irrigation and mild water deficit (75% FC) conditions, while under severe drought (25% FC), these treatments especially ZnO‐NPs intensified plant stress. CAT activity and proline content increased considerably in response to ZnO‐NPs 50 mg L^−1^ × MgSO_4_ 3 g L^−1^ under FC 75% and FC 25%. Application of ZnO‐NPs and MgSO_4_ effectively alleviated drought‐induced damages under moderate irrigation (FC 75%) and enhanced plant tolerance to water stress. Results of PCA and Cluster analysis proved that application of ZnO‐NPs at 50 mg L^−1^ + MgSO_4_ 3 g L^−1^ provide the most balanced improvement in growth, flowering, and stress tolerance traits under mild drought stress.

## Graphical abstract

ZnO nanoparticles and MgSO_4_ synergistically alleviated drought‐induced damage in saffron. Foliar application improved relative water content and antioxidant enzyme activity. Photosynthetic performance and membrane stability were enhanced under water deficit. Moderate drought combined with ZnO‐NPs and MgSO_4_ maximized saffron productivity.

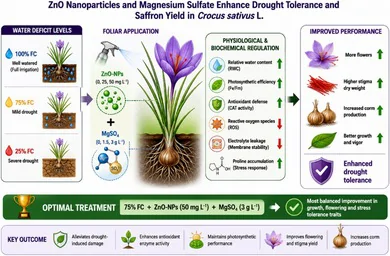

## 1 Introduction

*Crocus sativus* L. (Saffron), a marketable perennial geophyte plant, cultivates in Mediterranean climate, is a valued therapeutic plant, recognizing for the color, flavor, and extensive nourishing properties of its dried stigma (Marzabadi et al. 2021) Saffron product is one of the most appreciated, attractive, expensive, and extensively used flavors in the world (Leone et al. 2018; Siddique et al. 2020; Lachkar et al. 2022). About 150 biological constituents have been recognized in saffron stigma. The main bioactive constituents of saffron including safranal, crocin, and picrocrocin are primarily responsible for taste and pharmacologic assets of saffron (Khorasany and Hosseinzadeh 2016). The saffron farming commonly needs negligeable water, however inadequate irrigation can undesirably influence its yield and quality (Gusain and Joshi 2025). In a study, the yearly water necessity of saffron is stated about 288 mm (Latief Ahmad et al. 2017).

Drought is a key factor that enforces substantial reduction in plant production, resulting word food security. Drought destroys normal plant metabolism and processes, resulting declined growth and yield (Khan et al. 2025). Drought stress causes instabilities in plant water relations, imbalance ionic homeostasis, demolition of cell membranes, and photosynthesis reduction (Ali et al. 2025).

Agricultural scientists are responsively employed to improve plant production, simultaneously reducing plant water consumption, especially in drought‐prone areas through agronomical practices (Khan et al. 2025). Maximizing nutrient supplementation is an effective tool to achieve these goals (Shekhawat et al. 2021; Kumar and Sindhu 2024).

Zinc (Zn) as a trace micronutrient plays a crucial role in numerous physiological processes, growth regulation, and photosynthesis (Liu et al. 2025; Dang et al. 2024; Umair Hassan et al. 2020; Hamzah et al. 2022; Li et al. 2022; Veena and Puthur 2022; Nandal and Solanki 2021). Furthermore, Zn is involved in plant defense system through improvement in antioxidant enzyme activities under stress conditions and also encourages N uptake via regulating C–N metabolism (Guan et al. 2025).

Nano materials supplementation is a beneficial crop management technique in agriculture which are promising tools for alleviating drought stress (Singh et al. 2023, 2024). NPs scavenges the drought ‐mediated reactive oxygen species and preserves cell wall integrity (Singh et al. 2018) resulting improves plant tolerance to environmental stress (Maity et al. 2018; Nunes da Silva et al. 2022). ZnO–NPs a compatible and low‐toxic form of Zinc, employs as a proficient practical tool to enhance the tolerance of plants against stressful toxicity (Nedjimi 2025).

Magnesium (Mg^2+^) is one of the essential elements involves in plant growth, biochemical processes, and chlorophyll pigments (Pogłodzi'nski et al. 2021). Mg also regulates ribulose‐1,5‐bisphosphate carboxylase/oxygenase (Rubisco) activity, which is a key enzyme in the Calvin cycle (Muhammad Kaleem et al. 2025). Frequent studies have reported that Mg^2+^ has crucial role in adaption to abiotic stresses (Xuan et al. 2022; Farhangi‐Abriz and Ghassemi‐Golezani 2021). Even minor Mg^2+^ deficit impairs biochemical and physiological processes, resulting reduce biomass and plant sensitivity to external challenges (Senbayram et al. 2015).

We hypothesized that supplementation of Zinc Oxide nanoparticles (ZnO‐NPs) in combination with MgSO_4_ potentially can improve tolerance to drought stress as well as growth and flowering in saffron, through maintaining water status, enhancing antioxidant defense, preserving photosynthetic efficiency, and reducing membrane damage in saffron plants. Thus, we examined the effects of different levels of ZnO‐NPs in combination with different concentrations of Magnesium sulfate on growth, flowering, photosynthesis, and physio‐biochemical indicators under drought stress.

## 2 Material and Methods

In this study, saffron corms were planted in pots under research field conditions. The research was arranged in a RCBD using a split‐plot factorial experiment. Each experimental unit consists three replicates and each replicate three plants (nine plants); separately in one pot. The experiment was conducted during the 2023 and 2024 growing seasons at the University of Dhi Qar, Nasiriyah, Iraq. Uniform saffron corms were used to minimize variability among experimental units. The saffron ( *Crocus sativus* L.) corms used in this study were obtained from the University of Dhi Qar, Nasiriyah, Iraq. These corms are widely cultivated in the region, and their planting and propagation do not require any special permits from relevant authorities. The plant material was sourced and procured by the first author, Laila Turki Fadala, and was verified and approved for use in this research. The corms weighing 8–10 g were randomly cultivated at a depth of 10–12 cm in pots and the pots were randomly arranged in each plot.

Drought stress was imposed at three levels based on field capacity (FC), including 100% FC (well‐watered control), 75% FC (mild stress), and 25% FC (severe stress). FC was determined using the pot saturation–drainage method, where pots were fully saturated and allowed to drain freely for 48 h until gravitational water movement ceased. Soil moisture at this point was considered as FC, following the methodology described pervious (Kirkham 2014; Cassel and Nielsen 1986). Soil moisture content was monitored using a calibrated time domain reflectometry (TDR) device (Eijkelkamp, Netherlands), and irrigation was applied accordingly throughout the experimental period.

Three foliar applications were done included ZnO‐NPs at three concentrations (0, 25, and 50 mg L^−1^) and Magnesium Sulfate (MgSO_4_) at three concentrations (0, 1.5, and 3 g L^−1^). In the control treatment, distilled water was sprayed on the plants. Spraying was conducted three times with an interval of 10 days during active greening period (Late January to late March), which is the most golden opportunity for foliar feeding and transferring elements to the bulb (Ahmadi et al. 2025). Spraying was carried out using a 20‐l sprayer with a certain amount of water (based on 750 L/ha per spraying time) and fertilizer, along with the use of soap liquid as a surfactant, to increase the nutrient absorption capacity by the leaves. Each fertilizer was sprayed separately. When each fertilizer was sprayed, the sprayer was rinsed to remove the previous fertilizer residue, and then the new fertilizer was sprayed. The foliar spraying was done around 9 AM when the weather was sunny and calm, to maximize the absorption.

### 2.1 Soil Characterization

The soil texture was Clay‐Silt (0.82 ds m^−1^, pH 7.5) with organic matter content of 7.9 g kg^−1^ soil, total nitrogen (kjeldhal) of 0.06%, available phosphorus (P) of 12 mg^−1^ kg^−1^ soil, and available potassium (K) of 215 mg^−1^ kg^−1^.

### 2.2 ZnO‐NPs Characterization

The evaluation of different ZnO‐NP structures originated from US Research Nanomaterials Inc. (catalog no. US1019F; CAS no. 13463‐67‐7) was performed using various analytical techniques described in the literature (Ayadi et al. 2026). The morphology of the ZnO‐NPs were obtained with the Thermo Scientific Q250 Scanning Electron Microscope SEM. The size distribution of the ZnO‐NPs will be determined using the Malvern ZetaSizer Nano Particle Size Analyzer and the zeta‐potential (HPG) will be measured using a Malvern ZetaSizer Nano Particle Size Analyzer. The crystallographic phase of the ZnO‐NPs will be determined using an X‐ray diffraction (XRD) analysis using the Malvern PANalytical Xpert Pro instrument.

**FIGURE 1:**
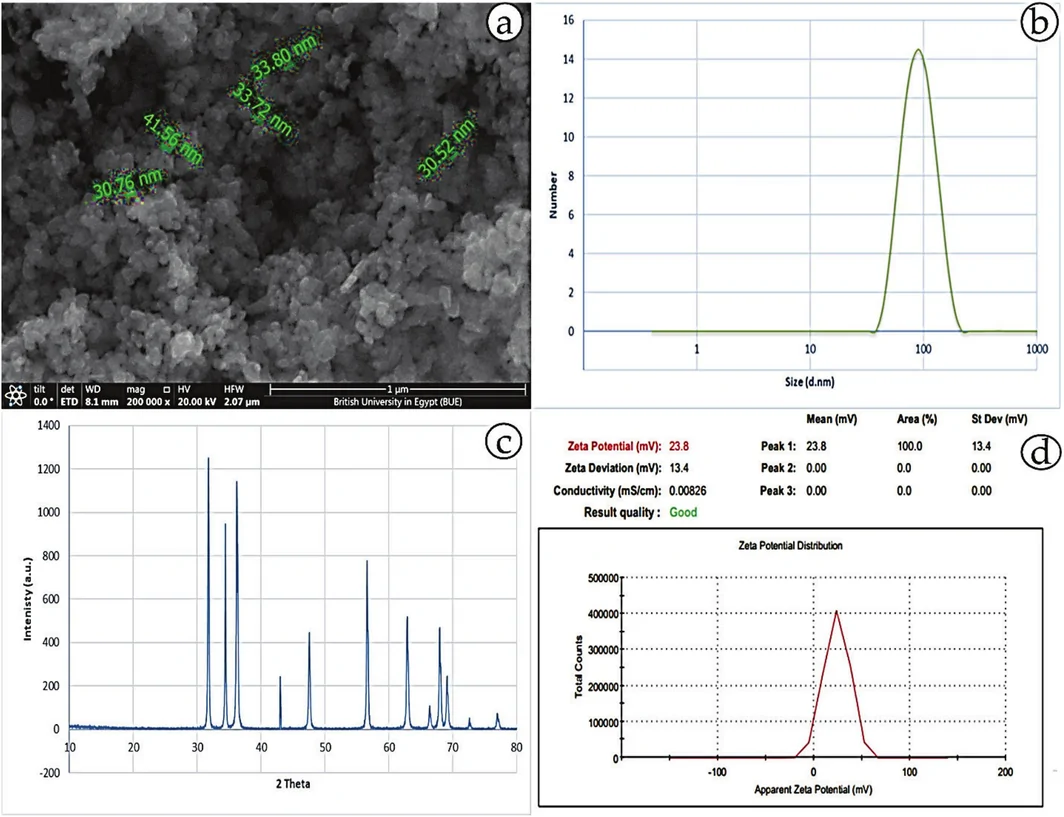
Examination of ZnO NPs: (a) SEM image, (b) Size distribution, (c) X‐ray diffraction analysis, and (d) Zeta potential.

The morphology of the ZnO nanoparticles was identified via FESEM images (Figure 1a), which clearly revealed that the morphological surface of the particles was spherical. Dynamic light scattering (DLS) revealed that the average size of the ZnO‐NPs was 91.3 nm, as shown in Figure 1b. XRD, which confirmed the crystalline phase of the nanoparticles, as shown in Figure 1c, and the XRD pattern of the sample revealed the formation of ZnO. Based on the comparison of their XRD patterns with the standard pattern of ZnO, they were quite identical to the characteristic peaks of the ZnO crystal. As for the ζ‐potential of 23.8 mV as measured via the electrophoretic light‐scattering technique, as shown in Figure 1d. ZnO‐NPs suspensions were carefully sonicated prior to each experiment to minimize the aggregation effect.

### 2.3 Measured Traits

#### 2.3.1 Leaf Dry Weight

To measure the leaf dry weight of saffron plants, fresh leaves were carefully detached and dried in an oven at 60°C–70°C for 48 h until complete moisture removal. The dried samples were then weighed using a precision balance with an accuracy of 0.001 g.

#### 2.3.2 Stigma Dry Weight

For stigma dry weight, fresh stigmas were carefully separated from flowers after harvest and dried in an oven at 40°C–50°C for 24–48 h to ensure complete dehydration. The dried stigmas were then weighed using a precision balance (accuracy of 0.001 g).

#### 2.3.3 Fresh Flower Weight

Fresh, healthy saffron flowers were collected immediately after harvest and weighed using a digital scale with an accuracy of 0.01 g to determine fresh flower weight.

#### 2.3.4 Flower Number

To count flower number, fully opened flowers per unit area (typically per square meter) were recorded during the peak flowering period. Daily field inspections were carried out, and the number of produced flowers was recorded over a defined period.

#### 2.3.5 Total Corm Weight

To assess total corm weight, healthy and intact corms were harvested, cleaned to remove soil and roots, and weighed using a precision balance with 0.01 g accuracy.

#### 2.3.6 Relative Water Content (RWC)

For determination of RWC, leaf discs (8 mm) were weighed fresh (WF), then soaked in distilled water for 2–3 h to obtain turgid weight (WT), and finally dried at 80°C for 24 h to record dry weight (WD) (Smart and Bingham 1974). RWC was calculated using: RWC = [(WF—WD)/(WT—WD)] × 100, indicating leaf hydration status.

#### 2.3.7 Electrolyte Leakage

Electrolyte leakage (EL), was estimated as: Five 8‐mm leaf discs from each treatment were placed in 15 mL of distilled water and gently agitated at room temperature for 24 h. The initial electrical conductivity (EC_2_) of the solution was then measured. Following this, samples were sterilized in an autoclave at 121°C for 30 min. Once cooled, the final conductivity (EC_2_) was determined (Lutts et al. 1995). The extent of ion leakage was calculated using the formula: EL = (EC_1_/EC_2_) × 100, representing the percentage of ions released from damaged cell membranes.

#### 2.3.8 Photosystem II Maximal Quantum Yield (F_V_ /F_M_ )

The maximum quantum yield of photosystem II (FV/FM) was estimated using the OJIP protocol as described in a recently paper (Habibi et al. 2024). Young mature leaves of saffron from were carefully selected for each experimental treatment. After 20 min in the dark‐adapted condition, chlorophyll fluorescence parameters were recorded using a portable fluorometer (Fluorpen FP 100‐MAX, Photon Systems Instruments, Drasov, Czech Republic).

#### 2.3.9 Proline Quantification

To assess proline levels, the protocol described by Bates et al. (1973) was adapted. Initially, 0.05 g of finely ground and dried plant tissue was mixed with 5 mL of a 3% sulfosalicylic acid solution and thoroughly homogenized using a porcelain mortar. The resulting homogenate was left to stand for 48 h before filtration. Then, a 1 mL aliquot of the filtrate was combined with 1 mL of ninhydrin reagent and 1 mL of glacial acetic acid. The mixture was incubated in a boiling water bath for 1 h. After allowing the samples to cool on ice, 2 mL of toluene was added, and the solution was vigorously shaken for 5 to 20 s. The upper toluene layer was separated and its absorbance was read at 520 nm to determine proline concentration.

#### 2.3.10 Catalase Activity

CAT activity was evaluated by tracking the decline in H_2_O_2_ absorbance at 240 nm over a 30‐s period (Dhindsa et al. 1981). The reaction mixture (3 mL total volume) included 50 mM phosphate buffer (pH 7.0), 15 mM hydrogen peroxide, and 100 μL of enzyme extract. The reaction began upon addition of H_2_O_2_, and absorbance was recorded immediately. Enzyme activity was expressed as the amount of catalase that breaks down 1 μmol of H_2_O_2_ per minute, using an extinction coefficient of 40 mM^−1^ cm^−1^ for calculation.

### 2.4 Statistical Analysis

Although the experiment was conducted over two growing seasons, only vegetative and reproductive traits were measured over 2 years, while other traits were assessed during a single growing season. Consequently, year effects were not analyzed, and the variables recorded in the second year were each analyzed as a distinct trait.

The analysis was performed using a factorial model based on a split‐factorial arrangement within a RCBD with three replications. Data analysis was conducted in R software (Ver. 4.5.1.). Prior to combined analysis, Levene's test for homogeneity of variances was conducted. A Three‐way variance analysis (ANOVA) was conducted to evaluate the effects of treatments at each sampling interval, with means compared using Tukey's HSD test at the 5% significance level. Principal component analysis (PCA) was applied to interpret the physiological response patterns. Additionally, the relationships among the evaluated traits were analyzed using the Pearson Correlation Coefficient and Cluster analysis were employed to identify relationships between variables and treatments.

## 3 Results

The analysis of variance (ANOVA) (Table 1) discovered some significant main effects and tow‐way and three‐way interactions for studied traits. Overall, Irrigation and ZnO‐NPs emerged as the most influential factors across the majority of agronomic and physiological traits, with several significant interaction effects indicating complex synergistic responses among nutrient treatments.

**TABLE 1:** The results of ANOVA for studied morpho‐physiological, physiological, biochemical, and flower yield traits of saffron under deficit irrigation, and foliar application of ZnO‐NPs and MgSo_4_.

Source	df	FY leaf DW	SY leaf DW	FY stem DW	SY stem DM	FY flower FW	SY flower FW	FY flower number
Rep	2	0.03^ns^	0.74^ns^	0.0014^ns^	0.004^ns^	3.42^ns^	60.11^ns^	55.15^ns^
FC	2	5.91***	83.57***	0.02*	0.047***	148.21***	12,600.11***	8205.48***
FC × *R*	4	0.25^ns^	3.95^ns^	0.003^ns^	0.005^ns^	6.80^ns^	5.59^ns^	111.87^ns^
ZnO‐NPs	2	0.72^ns^	6.58^ns^	0.011^ns^	0.006^ns^	13.9^ns^	194.04^ns^	265.04^ns^
MgSO_4_	2	0.34^ns^	8.4^ns^	0.003^ns^	0.005^ns^	7.38^ns^	62.11^ns^	358.48^ns^
FC × ZnO‐NPs	4	0.97^ns^	4.4^ns^	0.008^ns^	0.014^ns^	40.99^ns^	629.04**	100.52^ns^
FC × MgSO_4_	4	0.85^ns^	6.4^ns^	0.002^ns^	0.005^ns^	9.42^ns^	17.22^ns^	97.96^ns^
ZnO‐NPs × MgSO_4_	4	0.07^ns^	10.35^ns^	0.013*	0.013^ns^	49.15*	455.37*	410.96*
FC × ZnO‐NPs × MgSO_4_	8	0.6^ns^	4.76^ns^	0.007^ns^	0.011^ns^	34.77^ns^	390.56*	519.81**
Error	48	0.4	1.07	0.005	0.006	5.6	8.2	12.37
CV		15.79	19.5	1.25	1.33	26.12	27.96	23.52

Source	df	SY flower number	SY capitulum number	Catalase	Proline	Ionic leakage	RWC	Fv/fm
Rep	2	60.11^ns^	3.75^ns^	4.68^ns^	0.06^ns^	0.002^ns^	16.53^ns^	0.003^ns^
FC	2	12,600.11***	0.32^ns^	71.57***	1.93***	0.51***	60.81**	0.012***
FC × *R*		46.72^ns^	7.57^ns^	1.14^ns^	0.02^ns^	0.35^ns^	5.10^ns^	0.002^ns^
ZnO‐NPs	2	194.04^ns^	4.61^ns^	14.65*	1.60***	0.03^ns^	107.37***	0.01**
MgSO_4_	2	62.11^ns^	16.01*	10.11*	0.05^ns^	0.02^ns^	4.84^ns^	0.001^ns^
FC × ZnO‐NPs	4	629.04**	10.63^ns^	61.54***	1.51***	0.08*	29.65*	0.008***
FC × MgSO_4_	4	17.22^ns^	2.41^ns^	29.76***	0.76***	0.02^ns^	1.62^ns^	0.004^ns^
ZnO‐NPs × MgSO_4_	4	455.37*	2.73^ns^	82.49***	0.18^ns^	0.02^ns^	64.14***	0.002^ns^
FC × ZnO‐NPs × MgSO_4_	8	390.56*	2.51^ns^	15.08***	0.38***	0.05^ns^	11.56^ns^	0.003^ns^
Error	48	13.56	1.95	2.95	0.08	0.03	10.79	0.002
CV		21.14	28.86	11.4	3.49	4.21	12.3	0.25

*Note:* *, **, and ***, respectively significant at the levels of 0.04, 0.01, and 0.001.

Abbreviations: DM, dry matter; DW, dry weight; FW, fresh weight; FY, first year; ns, nonsignificant; SY: second year.

### 3.1 Fresh Flower Weight in First Year

**FIGURE 2:**
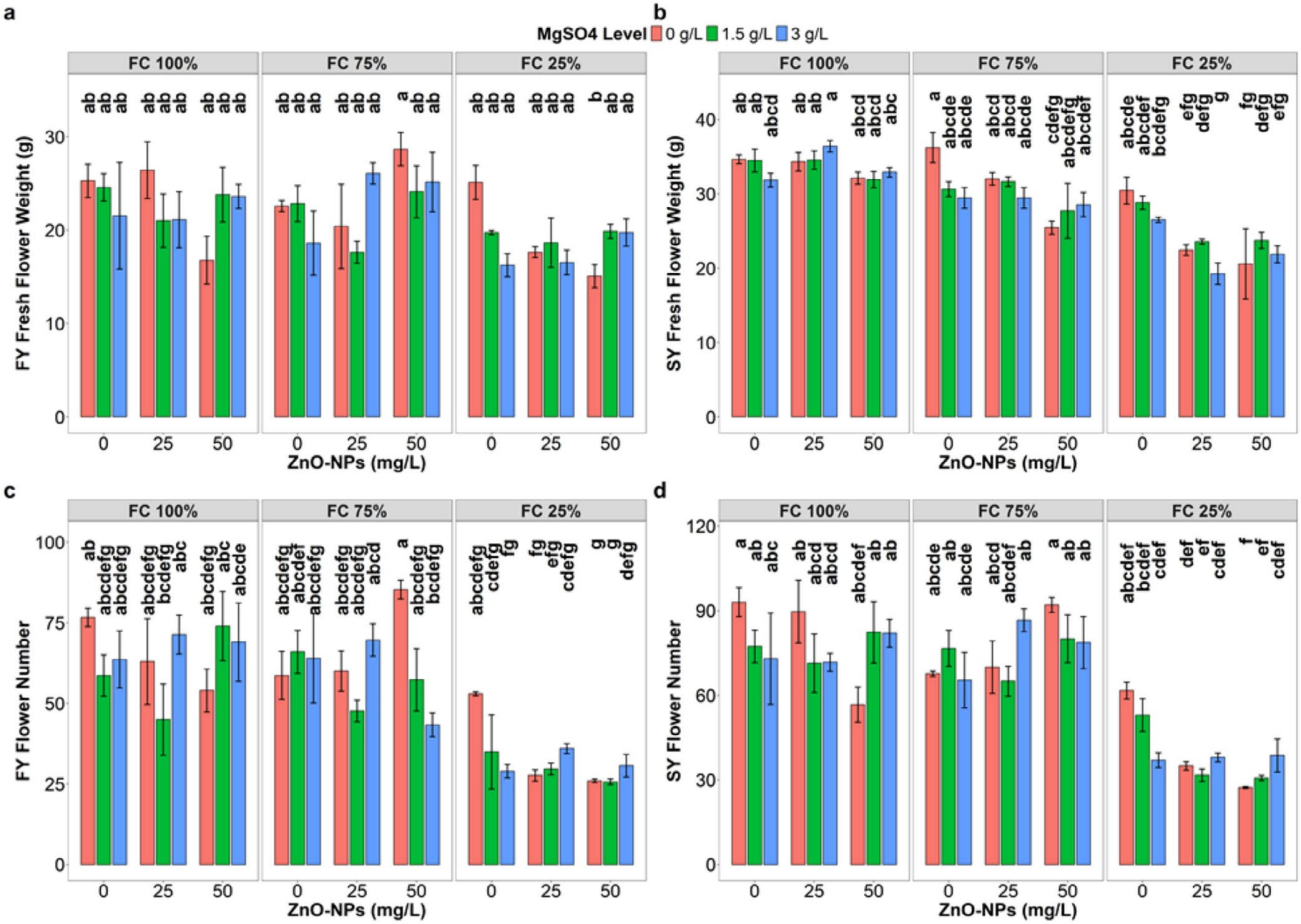
Effects of different irrigation levels and application of ZnO‐NPs and MgSO_4_ at different concentrations on flower‐related characteristics of Crocus sativus (Tukey’s HSD test, *r* = 3, *n* = 81). FY fresh flower weight (a), SY fresh flower weight (b), FY flower number (c), and SY flower number (d). FY: first year, SY: second year. Different lowercase letters above the bars indicate significant differences among treatment means according to Tukey’s HSD test at the 5% significance level (*p* < 0.05). Means sharing at least one common letter are not significantly different.

Fresh flower weight in the first year was significantly influenced by irrigation and foliar treatments. The highest value (28.68 g) was recorded under FC 75% irrigation and application of 50 mg L^−1^ ZnO‐NPs, while the lowest value (15.08 g) occurred under severe drought stress (FC 25%) and 50 mg L^−1^ ZnO‐NPs (Figure 2a). Under FC 75%, ZnO‐NPs 50 mg L^−1^ enhanced fresh flower weight by 54.1% compared with its value under ZnO‐NPs 25 mg L^−1^ × MgSO_4_ 1.5 g L^−1^. Under FC 25% Irrigation, application of ZnO‐NPs and MgSO_4_ slightly reduced the fresh flowers weight, so 50 mg L^−1^ ZnO‐NPs reduced flower weight by 66.5% relative to 25% FC control.

### 3.2 Fresh Flower Weight in the Second Year

The maximum fresh flower weight in second‐year (36.42 g) was obtained under FC 100% and application of 25 mg L^−1^ ZnO‐NPs +3 g L^−1^ MgSO_4_, whereas the minimum value (19.25 g) was recorded under severe drought stress (FC 25%) and application of 25 mg L^−1^ ZnO‐NPs +3 g L^−1^ MgSO_4_ (Figure 2b). Under FC 100%, ZnO‐NPs at 25 mg L^−1^ + MgSO_4_ at 3 g L^−1^ increased flower weight by 5.0% compared with control plants. Also, under severe water shortage (FC 25%), application of 25 mg L^−1^ ZnO‐NPs +3 g L^−1^ MgSO_4_ significantly reduced the flowers weight of second year by 58.2% compared to 25% FC control.

### 3.3 Flower Number in the First Year

The highest flower number in the first‐year (85.33) was achieved under FC 75% and application of 50 mg L^−1^ ZnO‐NPs, while the lowest value (25.67) was observed under FC 25% in response to 50 mg L^−1^ ZnO‐NPs +1.5 g L^−1^ MgSO_4_ (Figure 2c). Under FC 75%, ZnO‐NPs 50 mg L^−1^ significantly increased flower number by 48.8% compared with &50% FC control. However, under FC 25%, application of ZnO‐NPs at 50 mg L^−1^ + MgSO_4_ at 1.5 g L^−1^ caused a significant reduction by 106.4% in flower number.

### 3.4 Second‐Year Flower Number

The highest flower number (93.0 number) (Figure 2d) in second year was obtained under full irrigation condition (FC control), whereas the minimum value (27.3 number) was recorded under severe drought stress (FC 25%) and application of 50 mg L ZnO‐NPs. Under FC 100%, ZnO‐NPs at 50 mg L^−1^ reduced flower number by 64.1% and under FC 25%, decreased it by 125.6% compared to control.

Overall, results showed that application of ZnO‐NPs and MgSO_4_ under medium drought stress (75% FC) nonsignificantly improved flower production, while under severe drought stress significantly reduced it.

### 3.5 Leaf Dry Weight in First Year

**FIGURE 3:**
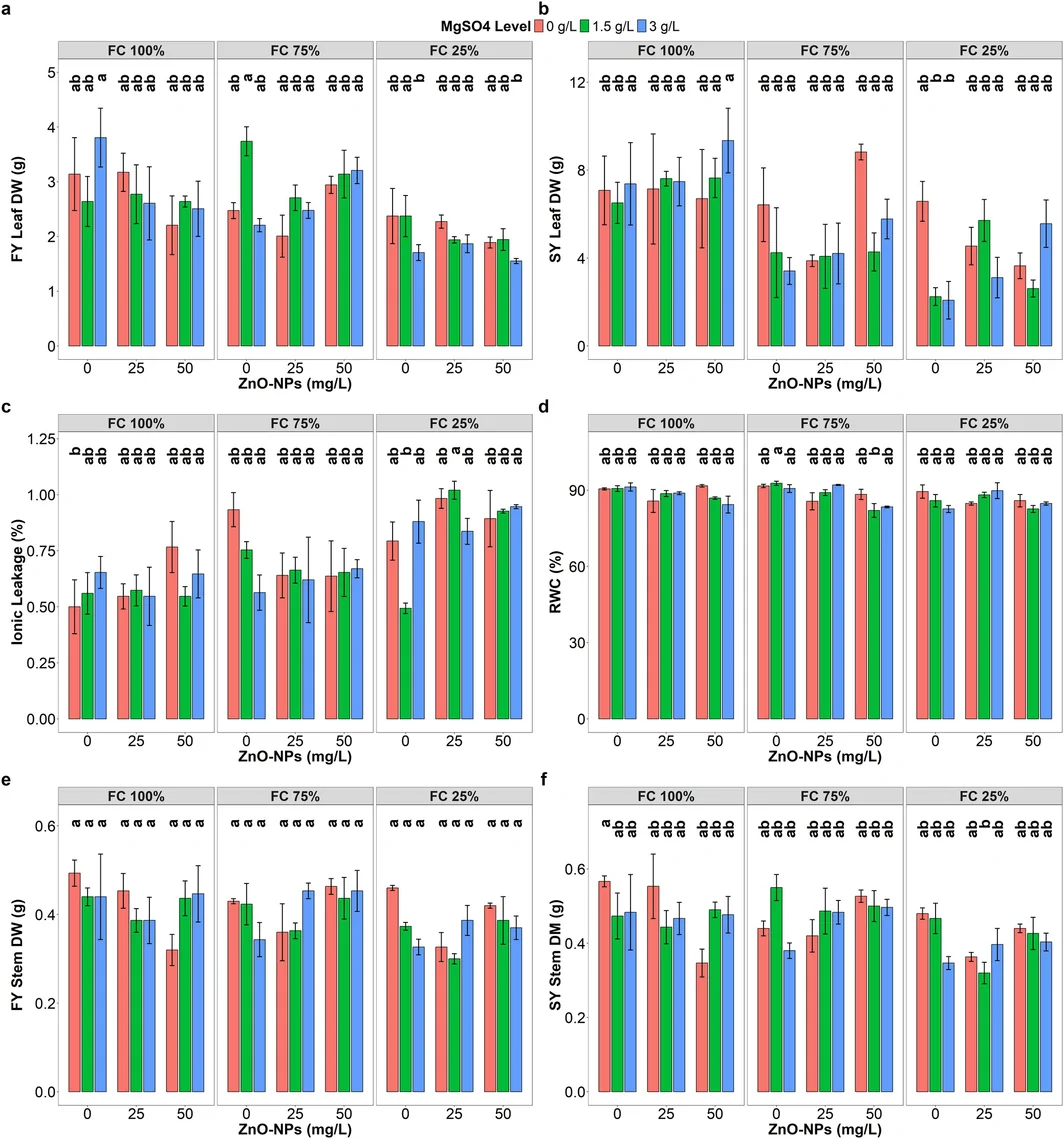
The morpho‐physiological and physiological characteristics, as well as stigma yield of *Crocus sativus* under different irrigation levels and application of ZnO‐NPs and MgSO_4_ at different concentrations (Tukey's HSD Test, *r* = 3, *n* = 81), Leaf dry weight in first year (a), leaf dry weight in second year (b), electrolyte leakage (c), relative water content (d), stigma dry weight in first year (e), and stigma dry weight in second year (f). FY, first year; SY, second year. Different lowercase letters above the bars indicate significant differences among treatment means according to Tukey’s HSD test at the 5% significance level (*p* < 0.05). Means sharing at least one common letter are not significantly different.

The highest leaf dry weight in the first year (3.81 g) was recorded under full irrigation condition and application of MgSO_4_ 3 g L^−1^, whereas the lowest value (1.55 g) was obtained under FC 25% and application of 50 mg L^−1^ ZnO‐NPs +3 g L^−1^ MgSO_4_ (Figure 3a). Severe drought stress (FC 25%) markedly reduced leaf dry weight compared with FC 100%. Under FC 25%, the combined treatment ZnO‐NPs 50 mg L^−1^ × MgSO_4_ 3 g L^−1^ decreased leaf dry weight by 52.6% compared to FC 25% control. Under FC 75%, application of MgSO_4_ at 1.5 g L^−1^ enhanced leaf dry weight by 51.9% compared with control. Moreover, under FC 100%, MgSO_4_ at 3 g L^−1^ increased leaf dry weight by 32.7% compared with control.

### 3.6 Leaf Dry Weight in Second Year

The maximum dry weight of leaves in second year (9.35 g) was obtained under FC 100% and application of ZnO‐NPs 50 mg L^−1^ + MgSO_4_ 3 g L^−1^, while the minimum value (2.08 g) was recorded under FC 25% and MgSO_4_ 3 g L^−1^ (Figure 3b). Under full irrigation, ZnO‐NPs 50 mg L^−1^ + MgSO_4_ 3 g L^−1^ improved leaf dry weight by 32.1% relative to control. Under FC 75%, ZnO‐NPs 50 mg L^−1^ enhanced leaf dry weight by 76.3% compared to control and 157.8% compared with plants treated with MgSO_4_ at 3 g L^−1^. Under severe stress (FC 25%), all treatment in combination or alone decreased leaf dry wight compared to control; ZnO‐NPs at 25 mg L^−1^ + MgSO_4_ at 1.5 g L^−1^ decreased leaf dry weight by 41.2% compared to control, while increased it abought 174.0% compared with plants treated with MgSO_4_ at 3 g L^−1^.

### 3.7 Electrolyte Leakage

Ionic leakage increased significantly under water deficit conditions. The highest ionic leakage (1.02%) was observed under FC 25% and application of ZnO‐NPs at 25 mg L^−1^ + MgSO_4_ at 1.5 g L^−1^, whereas the lowest value (0.49%) recorded under FC 25% + MgSO_4_ at 1.5 g L^−1^ (Figure 3c). Under full irrigation condition, application of ZnO‐NPs at 50 mg L^−1^ increased ionic leakage by 35.1% compared with control, Under FC 75%, MgSO_4_ 3 g L^−1^ decreased ionic leakage by 39.8% compared with 75% FC control. Finally, at FC 25%, application of MgSO_4_ at 1.5 g L^−1^ reduced ionic leakage by 51.9% compared to plants treated with ZnO‐NPs 25 mg L^−1^ + MgSO_4_ 1.5 g L^−1^, while increased it compared to control 25% FC.

### 3.8 Relative Water Content

RWC was significantly affected by irrigation and foliar application of MgSO_4_. The highest RWC (92.76%) was recorded under FC 75% + MgSO_4_ at 1.5 g L^−1^, while the lowest value (82.03%) occurred under FC 75% and application of ZnO‐NPs 50 mg L^−1^ + MgSO_4_ 1.5 g L^−1^ (Figure 3d) Under FC 100%, ZnO‐NPs at 50 mg L^−1^ increased RWC by 7.5% compared with those treated with ZnO‐NPs 50 mg L^−1^ + MgSO_4_ 3 g L^−1^. Under FC 75%, foliar application of MgSO_4_ 1.5 g L^−1^ improved RWC by 13.1% compared with plants which treated with ZnO‐NPs 50 mg L^−1^ + MgSO_4_ 1.5 g L^−1^. Under FC 25%, ZnO‐NPs at 25 mg L^−1^ + MgSO_4_ at 3 g L^−1^ enhanced RWC by 8.7% compared with control.

### 3.9 Stigma Dry Weight in the First Year

Stigma dry weights in the second year were ranged from 0.30 to 0.49 g. The highest value (0.49 g) was observed under FC 100%, whereas the lowest value (0.30 g) was recorded under FC 25% and application of ZnO‐NPs 25 mg L^−1^ + MgSO_4_ 1.5 g L^−1^ (Figure 3e) Under FC 25%, ZnO‐NPs 25 mg L^−1^ + MgSO_4_ 1.5 g L^−1^ reduced stigma dry weight by 53.3% compared with FC25% control.

### 3.10 Stigma Dry Weight in the Second Year

The highest stigma dry weight in the second year (0.57 g) was recorded under FC 100%, whereas the lowest value (0.32 g) was observed under FC 25% and application of ZnO‐NPs 25 mg L^−1^ + MgSO_4_ 1.5 g L^−1^ (Figure 3f). Under severe drought (FC 25%), foliar spraying of ZnO‐NPs 25 mg L^−1^ + MgSO_4_ 1.5 g L^−1^ significantly reduced stigma dry weight by 50.0% compared with FC25% (control).

### 3.11 Corm Number in the Second Year

**FIGURE 4:**
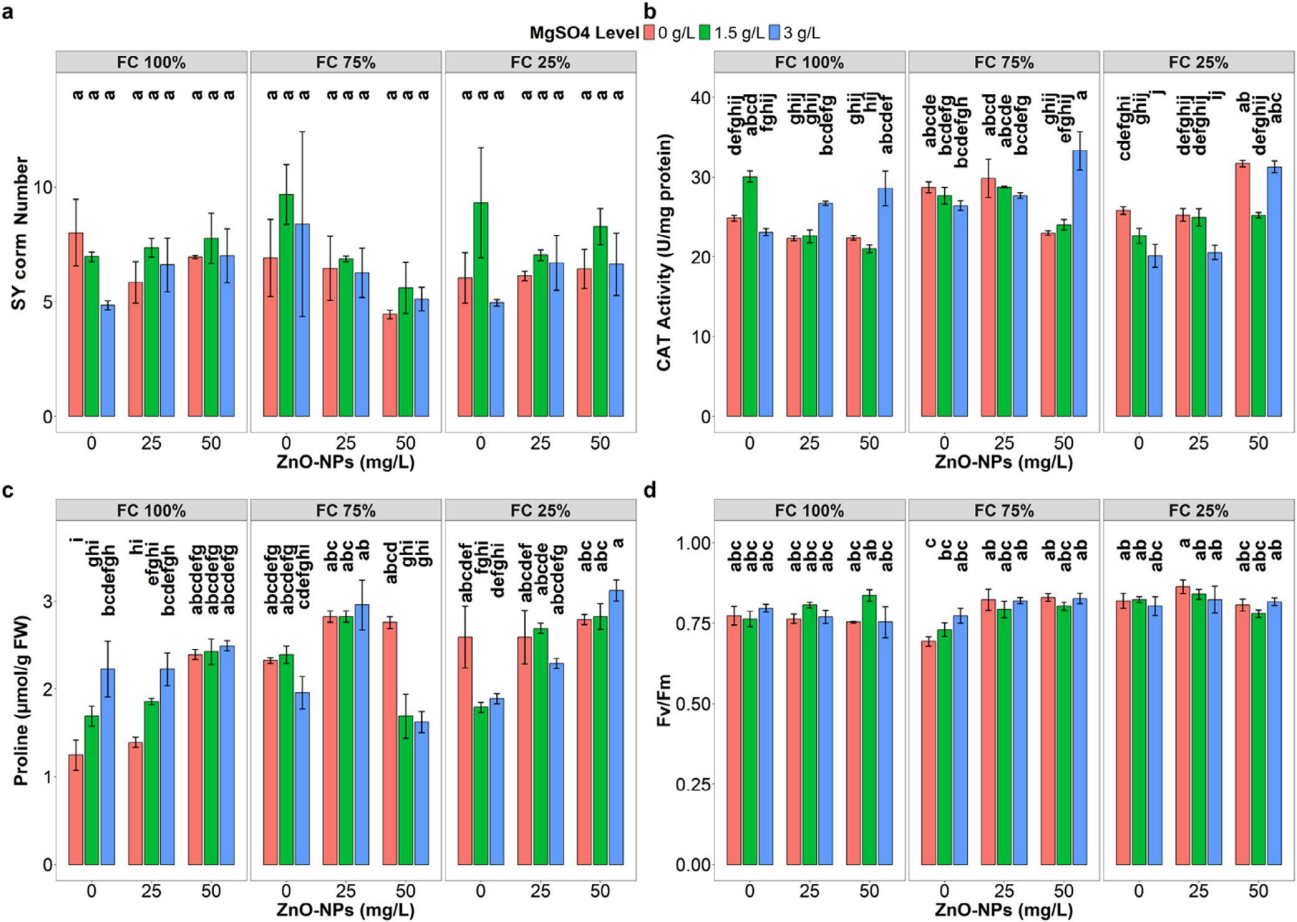
The corm production, catalase activity, and physiological attributes of *Crocus sativus* under different irrigation levels and application of ZnO‐NPs and MgSO_4_ at different concentrations (Tukey's HSD Test, *r* = 3, *n* = 81), corm number in second year (a), catalase activity (b), proline content (c), and Fv/Fm value (d). FY, first year; SY, second year. Different lowercase letters above the bars indicate significant differences among treatment means according to Tukey’s HSD test at the 5% significance level (*p* < 0.05). Means sharing at least one common letter are not significantly different.

In the second year after plantation, the numbers of corm varied from 4.45 to 9.68. The highest corm number (9.68) was recorded under FC 75% and application of MgSO_4_ at 1.5 g L^−1^, whereas the lowest value (4.45) occurred under FC 75% in response to ZnO‐NPs at 50 mg L^−1^ (Figure 4a). Under FC 75%, the corm numbers significantly were increased by 117.5% in plants treated with MgSO_4_ 1.5 g L^−1^ compared with those treated with ZnO‐NPs at 50 mg L^−1^. Under FC 25%, application of 1.5 g L^−1^ MgSO_4_ improved corm number by 88.1% compared with control.

### 3.12 Catalase Activity

Catalase activity was significantly affected by the interaction treatments. The highest CAT activity (33.29 U mg^−1^ protein) was observed under FC 75% and application of 50 mg L^−1^ ZnO‐NPs +3 g L^−1^ MgSO_4_, whereas the lowest value (20.12 U mg^−1^ protein) recorded under FC 25% and application of MgSO_4_ 3 g L^−1^ (Figure 4b). Under FC 75%, ZnO‐NPs 50 mg L^−1^ + MgSO_4_ 3 g L^−1^ enhanced CAT activity by 44.8% compared with plants that treated with ZnO‐NPs 50 mg L^−1^ and 21.6% compared to control. Under FC 25%, the CAT activity was improved in plants treated with ZnO‐NPs 50 mg L^−1^ by 57.5% compared with those treated with MgSO_4_ 3 g L^−1^ and by 24.76% compared to control (FC 25%).

### 3.13 Proline Content

The proline accumulation in the leaves significantly was increased under drought stress conditions. The highest proline content (3.12 μmol g^−1^ FW) was recorded under FC 25% and application of ZnO‐NPs 50 mg L^−1^ + MgSO_4_ 3 g L^−1^, while the lowest value (1.25 μmol g^−1^ FW) was observed in the control plants (FC 100%) (Figure 4c). Under severe drought stress (FC 25%), ZnO‐NPs at 50 mg L^−1^ + MgSO_4_ 3 g L^−1^ increased proline accumulation by 22.1% compared with 25% FC control. Under FC 75%, ZnO‐NPs 25 mg L^−1^ + MgSO_4_ 3 g L^−1^ enhanced proline content by 82.7% compared with plants that treated with MgSO_4_ 3 g L^−1^. Under FC 100%, ZnO‐NPs 50 mg L^−1^ + MgSO_4_ 3 g L^−1^ increased proline content by 99.2% compared with control.

### 3.14 Fv/Fm

The highest Fv/Fm value (0.86) was recorded under FC 25% and application of ZnO‐NPs 25 mg L^−1^, whereas the lowest value (0.69 value) was recorded under 75% FC irrigation. Under FC 25%, ZnO‐NPs 50 mg L^−1^ + MgSO_4_ 1.5 g L^−1^ decreased Fv/fm compared to control, while Under FC 75%, ZnO‐NPs 50 mg L^−1^ increased Fv/fm by 20.3% relative to control. Finally, under FC 100%, ZnO‐NPs 50 mg L^−1^ + MgSO_4_ 1.5 g L^−1^ enhanced Fv/fm by 12.0% compared with plants treated with ZnO‐NPs at 50 mg L^−1^ alone.

### 3.15 Principal Component Analysis

**FIGURE 5:**
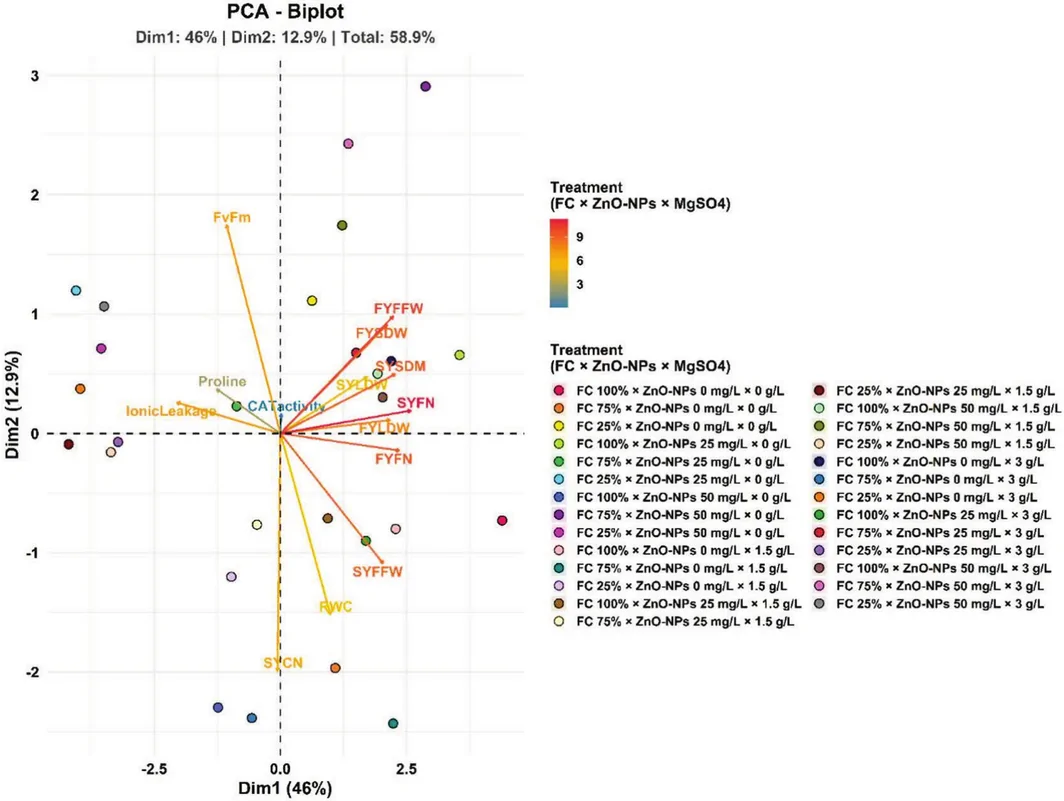
The PCA graph for studied variables of *Crocus sativus* under different irrigation levels and application of ZnO‐NPs and MgSO_4_ at different concentrations. FY, first year; FYFFW, first year flower fresh weight; FYFN, first year flower number; FYLDW, first year leaf dry weight; FYSDW, first year stigma dry weight; SY, second year; SYCN, second year corm number; SYFFW, second year fresh flower weight; SYFN, second year flower number; SYLDW, second year leaf dry weight; SYSDM, second year stigma dry weight.

PCA (Figure 5) revealed considerable variation among the irrigation, ZnO‐NPs, and MgSO_4_ treatments. The first two principal components explained 58.55% of the total variability, with PC1 and PC2 accounting for 45.54% and 13.01%, respectively. PC1 was strongly associated with growth and flowering traits, including flower number, stigma dry matter, fresh flower weight, and leaf dry weight. The second component was mainly related to stress‐response parameters such as CAT activity, proline accumulation, ionic leakage, and Fv/Fm, suggesting that stress‐related traits increased under drought conditions. The PCA biplot clearly separated treatments according to water availability and nutrient supplementation. Treatments receiving full irrigation (100% FC) generally occupied the positive side of PC1 and were associated with improved growth, higher flower production, and better physiological performance. Among these, the combination of full irrigation with ZnO‐NPs application showed particularly high Dim.1 values, indicating enhanced plant vigor and productivity. Conversely, treatments under severe drought stress (25% FC) were positioned on the negative side of PC1 and were characterized by reduced biomass, lower flower numbers, and increased stress indicators. PC2 mainly represented physiological adaptation traits. Fv/Fm, fresh flower weight, and stigma dry weight showed positive associations with PC2, while corm number and RWC exhibited strong negative relationships. Treatments supplemented with ZnO‐NPs and MgSO_4_ under moderate water stress tended to shift toward more favorable PCA positions, suggesting partial alleviation of drought stress. Full irrigation generally caused better flowering performance, lower ionic leakage, and improved physiological stability. In contrast, severe drought reduced growth and increased stress responses. Application of ZnO‐NPs and MgSO_4_ improved several growth and physiological traits even under reduced irrigation (75% FC), indicating their beneficial role in enhancing drought tolerance.

### 3.16 Pearson's Correlation Analysis

**FIGURE 6:**
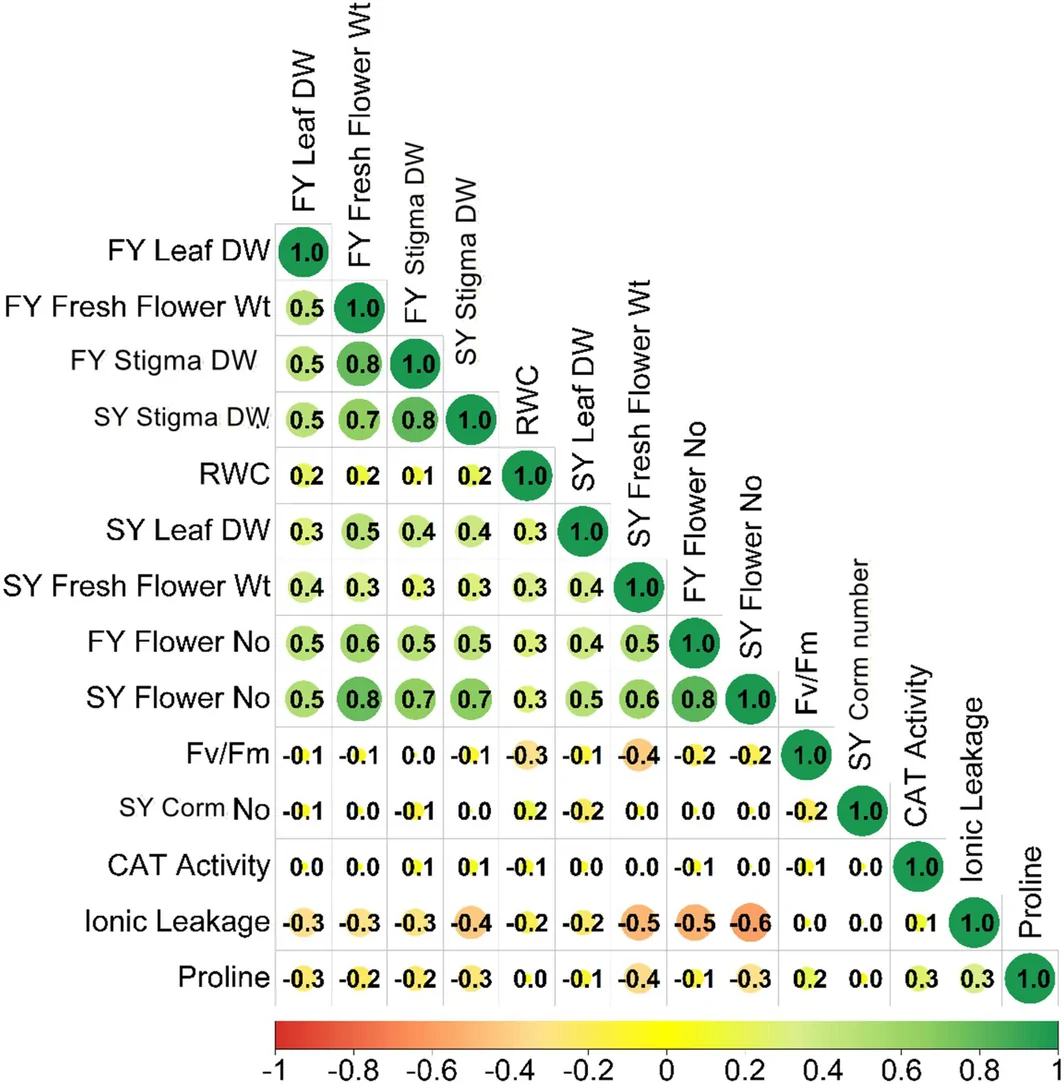
The Pearson's correlation matrix for studied variables of *Crocus sativus* under different irrigation levels and application of ZnO‐NPs and MgSO_4_ at different concentrations. FY, first year; SY, second year.

Pearson correlation analysis (Figure 6) revealed strong interrelationships among vegetative growth, flowering traits, and physiological parameters under the different irrigation and nutrient treatments. In general, most growth and yield‐related traits exhibited positive correlations with each other, whereas ionic leakage and proline accumulation showed predominantly negative relationships with plant productivity traits. leaf dry weight exhibited moderate to strong positive correlations with stigma dry weight (*r* = 0.476), fresh flower weight (*r* = 0.475), and flower number (*r* = 0.519). The strongest positive association was observed between flower number in first year and flower number in second year (*r* = 0.817). These results suggest a close functional relationship between plant structural growth and flower productivity. In addition, fresh flower weight showed strong positive correlations with stigma dry weight (*r* = 0.784), demonstrating that vigorous stigma development supported greater floral biomass accumulation. Ionic leakage displayed consistent negative correlations with most growth and flowering traits. The strongest negative relationships were detected between ionic leakage with flower number (*r* = −0.584) and fresh flower weight (*r* = −0.499). These findings indicate that increased membrane damage adversely affected plant growth and reproductive efficiency. Proline accumulation was negatively correlated with most vegetative and reproductive traits, including fresh flower weight (*r* = −0.358), flower number (*r* = −0.323), stigma dry matter (*r* = −0.274), and leaf dry weight (*r* = −0.252). However, proline showed a positive correlation with ionic leakage (*r* = 0.330) and CAT activity (*r* = 0.256), indicating that proline accumulation increased in response to oxidative and membrane stress conditions.

### 3.17 Cluster Classification

**FIGURE 7:**
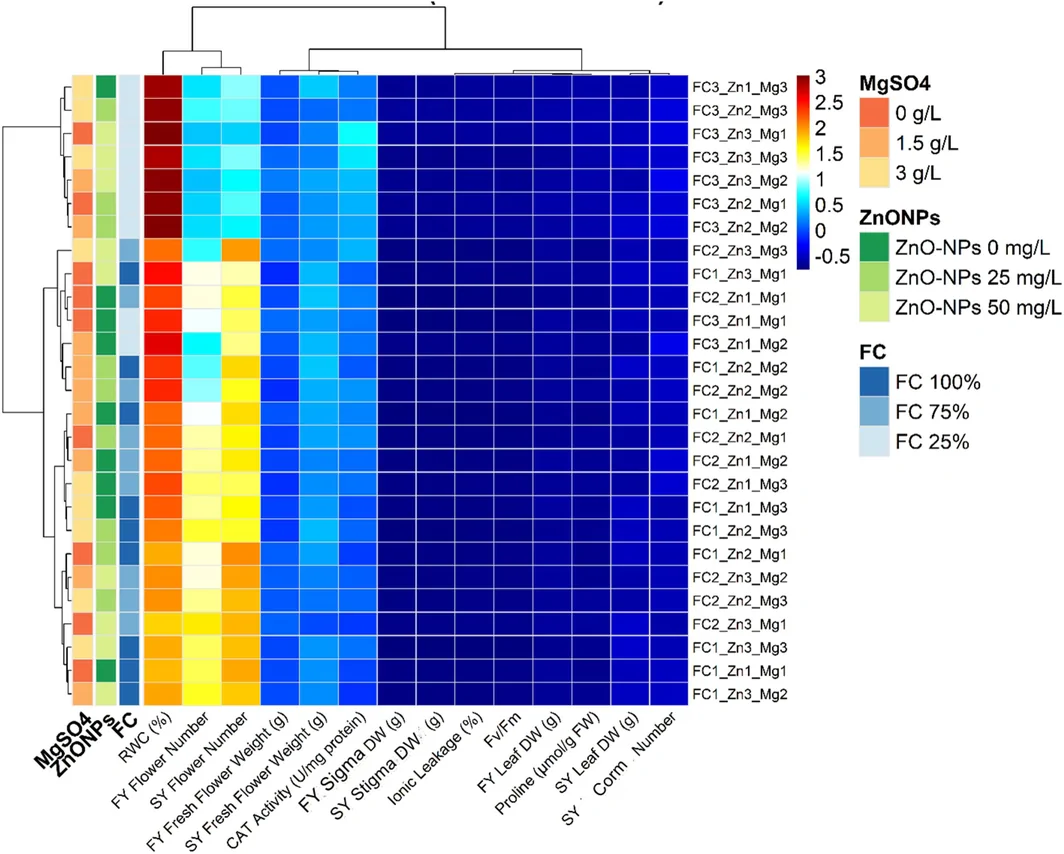
The cluster graph for studied variables of *Crocus sativus* under different irrigation levels and application of ZnO‐NPs and MgSO_4_ at different concentrations. FY, first year; SY, second year.

The classification analysis revealed clear multivariate patterns among the irrigation regimes and combined ZnO‐NPs × MgSO_4_ treatments (Figure 7). Treatments under severe water deficit (FC 25%) were generally separated from FC 75% and FC 100% treatments due to their greater association with osmotic adjustment and antioxidant defense traits. The combined treatment FC 25% × ZnO‐NPs 50 mg L^−1^ × MgSO_4_ 3 g L^−1^ (FC3_Zn3_Mg3) showed high positive loading values for CAT activity (0.580), indicating strong activation of the antioxidant defense system under severe drought stress. This treatment was also associated with elevated proline accumulation and improved osmotic regulation, confirming its role in stress tolerance enhancement. Likewise, FC 25% × ZnO‐NPs 50 mg L^−1^ × MgSO_4_ 0 g L^−1^ (FC3_Zn3_Mg1) and FC 25% × ZnO‐NPs 25 mg L^−1^ × MgSO_4_ 0 g L^−1^ (FC3_Zn2_Mg1) exhibited relatively high CAT activity coefficients (0.679 and 0.380, respectively), suggesting that ZnO‐NPs application under water deficit stimulated enzymatic antioxidant responses. The FC 25% treatments were generally characterized by higher RWC coefficients, particularly FC 25% × ZnO‐NPs 50 mg L^−1^ × MgSO_4_ 0 g L^−1^ (3.025), FC 25% × ZnO‐NPs 25 mg L^−1^ × MgSO_4_ 1.5 g L^−1^ (3.012), and FC 25% × ZnO‐NPs 25 mg L^−1^ × MgSO_4_ 0 g L^−1^ (2.965). These results indicate that ZnO‐NPs and MgSO_4_ combinations partially alleviated the negative effects of drought stress by maintaining plant water status. In contrast, FC 100% irrigation treatments were more closely associated with vegetative growth and reproductive performance. The treatment FC 100% × ZnO‐NPs 0 mg L^−1^ × MgSO_4_ 0 g L^−1^ (FC1_Zn1_Mg1) exhibited high coefficients for first‐ and second‐year flower number (1.45 and 1.92, respectively), indicating improvement in flowering performance under optimal irrigation conditions. Similarly, FC 100% × ZnO‐NPs 50 mg L^−1^ × MgSO_4_ 3 g L^−1^ (FC1_Zn3_Mg3) showed strong associations with flowering traits, especially second‐year flower number (1.83), suggesting that the combined application enhanced reproductive capacity under nonstress conditions. Moderate irrigation stress (FC 75%) combined with ZnO‐NPs and MgSO_4_ treatments produced balanced responses between growth and stress tolerance. For instance, FC 75% × ZnO‐NPs 50 mg L^−1^ × MgSO_4_ 0 g L^−1^ (FC2_Zn3_Mg1) exhibited high coefficients for first‐year flower number (1.68) and second‐year flower number (1.87), while maintaining relatively low stress‐related coefficients. This indicates that moderate drought combined with ZnO‐NPs may optimize resource allocation between vegetative growth and flowering. The clustering pattern further demonstrated that FC 25% treatments formed a distinct group characterized by elevated CAT activity and proline accumulation, whereas FC 100% and FC 75% clustered according to higher growth, flowering, and biomass production. The close proximity among FC 75% × ZnO‐NPs 50 mg L^−1^ × MgSO_4_ 0–3 g L^−1^ suggests that these combinations had similar physiological and growth responses, indicating consistent drought mitigation effects.

## 4 Discussion

The results obtained showed that water deficit stress caused substantial changes in the vegetative growth, flowering activity, physiological parameters, and biochemical properties of saffron plants. Severe drought stress (FC 25%) resulted in a decrease of leaf dry weight, flower weight, flower number, stigma dry weight, and corm yield, while the ionic leakage increased and proline accumulation took place. Also, the enhanced catalase activity and accumulation of proline during the stress process indicated the activation of oxidative stress protection mechanisms and osmotic regulation processes in *Crocus sativus* plants. The elevated activity of catalase caused by the addition of ZnO‐NPs 50 mg L^−1^ + MgSO_4_ 3 g L^−1^ consumes excessive reactive oxygen species giving an opportunity for the adaptation to stress.

Water deficit decreases stomatal conductance and CO_2_ availability, leading to reduced carbohydrate production and lower biomass accumulation. Consequently, saffron plants under severe drought allocate available resources toward survival and maintenance processes rather than flower and stigma production. Water shortage closed the stomata, resulting reduces gaseous exchange, CO_2_ availability transpiration rate and photosynthesis, (Dirie et al. 2024; Raza et al. 2013). Additionally, drought stress deeply shrinkages cell wall stability and cell membrane integrity, subsequently raises high levels of oxidative stress which decrease plants growth (Ali et al. 2025).

In present study, the application of ZnO‐NPs and MgSO_4_ moderated the adverse effects of drought stress depending on the applied concentration under mild drought stress. Zinc activates more than 300 plant enzymes involves in the synthesis of chlorophyll, auxin metabolism, carbohydrate metabolism and maintenance of membrane integrity (Dubey and Chandra Pathak 2024; Mapodzeke et al. 2021). Zn up‐regulated the expression of aquaporin genes (MdPIP1;1, MdPIP1;2), increasing leaf RWC and promoted the transport of photosynthetic products from the leaves to roots. the activity of various ZIP genes is administered by transcription factors from the basic leucine zipper (bZIP) family, which are linked to the Zn deficiency response element (ZDRE) which are liable to Zn deficiency (Harborne 1973; Guan et al. 2025). It is probable that ZnO‐NPs lead to positive effects through their function in activating certain enzymes, inducing chlorophyll formation, stabilizing membranes, and boosting antioxidant behaviors. Similarly, MgSO_4_ has the ability to enhance chlorophyll production through regulation of stomata and osmotic processes. However, one must exercise caution when using ZnO‐NPs and MgSO_4_ due to the fact that these compounds may have an adverse effect when their concentration in the nutrient solution exceeds certain levels.

It could be inferred that the increase in RWC after the use of ZnO‐NPs could have been contributed by enhanced water uptake and movement. It has been known that zinc regulates aquaporin expression and increases root water permeability, which helps in maintaining tissue hydration. The higher values of Fv/fm in plants treated by ZnO‐NPs show enhancement of PSII protection from drought‐induced photoinhibition. The maintenance of PSII functioning helps to sustain photosynthesis and energy production under unfavorable conditions.

Magnesium has a key role in chlorophyll biosynthesis, chloroplast process, and photosynthesis (Masuda 2008; Hauer‐Jákli and Tränkner 2019). Mg can develop the leaf extension, resulting improves photosynthesis performance and carbohydrate biosynthesis, which lead to the enhanced plant growth (Abo El‐Ezz et al. 2024). Application of Mg as MgO‐NPs has enhanced photosynthetic performance, chlorophyll fluorescence, antioxidant defense, and growth of *Coriandrum sativum* under drought stress, resulting improves drought resilience (Muhammad Kaleem et al. 2025).

Magnesium is a vital nutrient necessary for plants' metabolism that is vital to their functioning, and when magnesium is deficient, its deficiency worsens drought stress conditions. In celery plants, Mg has allowed the ions to maintain their balance, especially when it comes to the most important inorganic ions such as ions of K, Mg, and Ca, which serve to maintain the turgor and overall metabolism in the cells (Gao et al. 2023). Besides being a part of the chlorophyll structure, magnesium is also an enzyme activator that is engaged in the process of photosynthesis and carbohydrate metabolism. Such an increase in photosynthesis may be the reason for the rise in dry leaf weight, flowering rate, and corm formation under moderate drought conditions. Magnesium is also responsible for the stabilization of the chloroplasts membranes, which allows for better physiological performance in the conditions of deficit water.

The cause of EL has been widely accepted as a symptom of membrane damage during stressful environmental conditions. The increase in EL due to extreme drought conditions is believed to be caused by disruption of membrane integrity by oxidative stress and lipid peroxidation. Lower EL values observed in some ZnO‐NPs and MgSO_4_ treatments suggest stabilization of membranes and possible enhancement of the protective antioxidant action of cells against reactive oxygen species. Overall, the results support the beneficial effects of Zn and Mg in cellular integrity during drought stress (Veena and Puthur 2022).

The increase in the amount of proline and catalase activity demonstrates important adaptive processes to mitigate the effects of drought. Proline acts as an osmoprotectant and provide a protection for proteins and cell membranes, as well as to eliminate free radicals. Additionally, catalase is a vital enzyme that degrades H_2_O_2_ in stressed plants, according to Ayadi et al. (2026). The increase in enzyme activity in plants treated with ZnO NPs and MgSO_4_ indicates the greater effectiveness of plants in performing antioxidant mechanisms in the case described above.

Interestingly, even though ZnO‐NPs induced enhanced antioxidant responses in stressful drought conditions, the increased CAT activity and proline accumulation were not always associated with greater flower production or biomass accumulation. It means that activation of the stress‐defense mechanisms requires considerable metabolism expenses. Therefore, depending on the situation, in condition of severe water shortage, plants may allocate funds for the survival matter instead of reproduction. Also, high nanoparticles concentrations may bring in certain additional physiological troubles in water‐shortage conditions (Coşkun et al. 2025).

Drought stress disrupts plant metabolism by inducing cellular dehydration, oxidative damage, nutrient imbalance, and impaired carbon assimilation, ultimately leading to substantial reductions in vegetative growth and reproductive productivity (Qiao et al. 2024; Ayadi et al. 2026; Ramzan et al. 2026). Among mineral nutrients, zinc (Zn) and magnesium (Mg) play complementary physiological roles in enhancing drought tolerance by maintaining redox homeostasis, protecting the photosynthetic apparatus, and sustaining reproductive development (Umair Hassan et al. 2020; Sarraf et al. 2026; Mandal et al. 2026; Joseph et al. 2026). Their coordinated effects explain the significant improvements frequently observed in growth, yield, and seed quality under water‐deficit conditions. One of the primary consequences of drought is the excessive accumulation of reactive oxygen. Although ROS function as signaling molecules at low concentrations, excessive accumulation causes lipid peroxidation, protein oxidation, membrane disruption, and nucleic acid damage (Hong et al. 2024). Zinc indirectly suppresses oxidative stress by stabilizing membrane phospholipids and sulfhydryl groups of proteins while acting as a structural or regulatory component of numerous antioxidant enzymes (Lee 2018; Marreiro et al. 2017).

Zinc contributes to the functioning of superoxide dismutase (Cu/Zn‐SOD), catalase (CAT), ascorbate peroxidase (APX), and glutathione reductase (GR) and in addition to the functioning of enzymes taking part in detoxification of reactive oxygen species (Al‐Zahrani et al. 2021). Concurrently, magnesium is responsible for protecting chloroplasts and minimizing the effects of photooxidation via effective use of absorbed light energy (Ye et al. 2019; Jamali Jaghdani et al. 2021). In this way, an appropriate amount of zinc and magnesium results in lower amounts of malondialdehyde (MDA), hydrogen peroxide, and leakage of electrolytes in plants, which indicates a lower level of damage than when compared to non‐treated plants in drought. In addition to the activity of enzymatic antioxidants, zinc and magnesium are involved in the activity of nonenzymatic antioxidants.

Zinc triggers the biosynthesis of phenolic compounds, flavonoids, glutathione, and ascorbic acid by activating secondary metabolic pathways (Tian et al. 2025; Trevisan et al. 2014) while Mg supplies metabolic energy for synthesis and recycling of antioxidants due to the necessity of Mg‐ATP complexes in ATP‐based reactions (Barbagallo et al. 2023; Kröse and de Baaij 2024). Simultaneous boosting of enzymatic and nonenzymatic antioxidants increases the capacity of cellular redox buffering and gives the possibility for plants to continue functioning biologically even at very low water supply. Recently conducted molecular studies show the presence of the regulation of drought‐responsive signaling pathways in case of Zn and Mg. Zinc is engaged in zinc‐finger transcription factors, MAP kinase signaling, and abscisic acid (ABA) mediated stress‐response pathways that control redox and osmolyte biosynthesis, as well as processes of stomatal closure (Yin et al. 2025; Moulick et al. 2022). Magnesium is involved in retrograde signaling from chloroplast to nucleus, phosphorylation of proteins with the use of ATP, and carbohydrate signaling systems. These regulatory mechanisms make it possible to coordinate plant physiological responses at the whole‐plant level as a part of water‐saving strategies, mechanisms of oxidation protection, and reproductive stability.

Using PCA, it was shown that use of ZnO‐NP and MgSO_4_ has a positive effect on several growth and physiological traits under full irrigation and even at lower irrigation rates (75% FC), thus confirming their beneficial role in enhanced drought tolerance. The correlation analysis supported PCA conclusions by showing that the growth and flowering indicators were interrelated through stressful condition experienced by the plants. The positive relationships between leaf biomass, stigma dry matter, flower weight, and flower quantity indicate that the positive effect of treatments on vegetative growth translated to higher reproductive performance. At the same time, the increase in ionic leakage and proline accumulation indicates a loss due to stress and reduced crop yield.

Overall, the findings indicate that ZnO‐NPs and MgSO_4_ function primarily as physiological modulators that improve drought adaptation through maintenance of water relations, enhancement of antioxidant defenses, and preservation of photosynthetic performance. However, their effectiveness depends strongly on drought intensity, with the greatest benefits observed under moderate rather than severe water deficit conditions.

## 5 Conclusion

The interaction between irrigation regimes and ZnO‐NPs × MgSO_4_ treatments significantly influenced some morphological, physiological, and biochemical traits. Severe drought stress (FC 25%) substantially decreased vegetative growth, flower production, and corm formation, while increasing membrane damage and proline accumulation. However, foliar application of ZnO‐NPs and MgSO_4_ alleviated many negative effects of drought, under mild stress condition. In most cases, FC 75% combined with ZnO‐NPs 50 mg L^−1^ and MgSO_4_ 1.5–3 g L^−1^ produced favorable responses in growth, flowering, catalase activity, and photosynthetic efficiency. Therefore, the combined use of ZnO‐NPs and MgSO_4_ can be considered an effective strategy for improving drought tolerance and maintaining physiological properties under reduced irrigation conditions. The PCA demonstrated that water deficit stress markedly reduced biomass accumulation and reproductive performance, while increasing stress‐related traits such as ionic leakage and proline content. However, under slight drought condition, the combined application of ZnO‐NPs and MgSO_4_ improved plant tolerance to drought stress by enhancing physiological efficiency, maintaining growth, and promoting flowering. The Pearson correlation analysis demonstrated that enhanced vegetative growth strongly supported flower production and biomass accumulation.

## Author Contributions

**Seham Zaben:** investigation, conceptualization, validation, methodology, data curation. **Laila Turki Fadala:** project administration, methodology, supervision, conceptualization. **Heidar Meftahizade:** writing – review and editing, writing – original draft, software, formal analysis, supervision. **Ekhlas Meteab Ahmed Marir:** resources, supervision, methodology, visualization, software.

## Funding

The authors have nothing to report.

## Ethics Statement

This study is not a clinical trial and no human participants involved in this research.

## Consent

The authors declare their consent to the publication of this article.

## Conflicts of Interest

The authors declare no conflicts of interest.

## Data Availability

The data that support the findings of this study are available from the corresponding author upon reasonable request.
